# Editorial: Flagellar Motors and Force Sensing in Bacteria

**DOI:** 10.3389/fmicb.2022.833011

**Published:** 2022-02-04

**Authors:** Matthew A. B. Baker, Seiji Kojima, Ashley L. Nord, Jonathan D. Partridge

**Affiliations:** ^1^School of Biotechnology and Biomolecular Sciences (BABS), University of New South Wales, Sydney, NSW, Australia; ^2^Division of Biological Science, Graduate School of Science, Nagoya University, Nagoya, Japan; ^3^Centre de Biochimie Structurale de Montpellier, Montpellier, France; ^4^University of Texas at Austin, Austin, TX, United States

**Keywords:** flagellar, motility, bacteria, force sensing, archaea

The machinery behind bacterial flagellar motility is an astounding example of natural nanotechnology. The bacterial flagellar motor (BFM) is the end point of chemotaxis, a sensory pathway that helps bacteria to navigate their surroundings in search of an optimally hospitable niche. The BFM, which coordinates the rotation of an extracellular filament to drive motility is sensitive to the electrochemical and mechanical nature of its environment, dynamically tuning its structural composition and power output on the fly. For example, the BFM recruits torque-producing stator units to increase its power output in response to increased mechanical loads. Thus, the motor not only generates force and torque, but is also involved in surface sensing and mechanosensing. Bacterial motility underlies the virulence of pathogens, especially those which infect areas under high flow, as well as the linkage between force sensing, quorum sensing, and biofilm formation. Bacterial motility and surface interactions are a key area of study in the face of rising antimicrobial resistance, which is one of the dominant public health issues of this century. This Research Topic sought to highlight the similarities and diversities of the BFM and chemotaxis systems across species, to understand their evolutionary origins, and to reveal how the BFM's dynamic structure and activity can be modulated by, and in response to changes in its environment.

Many of the articles in this special issue highlight the diversity in both structure and assembly through investigations of traditionally less well-characterized flagellar motors. Zhou and Roujeinikova review the structure and composition of the proteinaceous periplasmic stator scaffolds found in many polar flagellar motors, imaged via electron cryotomography, which are instrumental for sustaining the abnormally high speeds and torques of monotrichous bacteria (as compared to peritrichous bacteria). Arroyo-Pérez and Ringgaard asked how *Vibrio parahaemolyticus* ensures proper positioning of its single flagellum at the old cell pole after cell division. They investigated the subcellular spatiotemporal localization of the polar flagellar determinants FlhF and FlhG, revealing their cell cycle-dependent intracellular coordination, which depends upon the cell pole determinant protein HubP and another still unidentified factor. Xu et al. investigated the mechanism by which the hook penetrates the peptidoglycan (PG) sacculus in spirochetes, providing evidence that FlgJ and BB0259, a previously uncharacterized cell wall-degrading enzyme, coordinate PG penetration in the Lyme disease spirochete *Borrelia burgdorferi*. Ferriera et al. shed light upon how different flagella evolved in closely related lineages. By contrasting data from protein phylogenetics and structural data from electron cryo-tomography and subtomogram averaging, they posit that Enterobacteriaceae flagella were horizontally acquired from an ancestral β-proteobacterium, giving them a ”general-purpose” BFM which is able to adjust to a wide range of conditions.

Other articles in this series spotlight the diverse ways in which bacteria adapt their behavior in response to external cues. Bacteria sense and adapt to physical forces in their environment, such as shear-induced or surface-induced forces on the membrane. Kenney reviews the ways in which histidine kinases funnel diverse environmental stimuli, including mechanical stress and changes in membrane tension, into changes in gene expression, underscoring that many of the molecular mechanisms underlying mechanical force sensing in bacteria remain to be unraveled. The activity of the BFM is also modulated by external cues, *via* a growing list of proteins and regulators, including the chemotaxis response regulator CheY and c-di-GMP-binding proteins that act as molecular brakes or clutches. In *Shewanella putrefaciens*, Pecina et al. identify MotL as a novel stand-alone PilZ-domain protein which regulates the lateral flagellar system in response to intracellular levels of the secondary messenger c-di-GMP. Exceptionally, MotL does not act upon the polar motor, thus demonstrating differential regulation of two flagellar systems in a single species. The soil motile *Azospirillum brasilense* has a complex chemotaxis system, with two distinct chemotaxis pathways and four CheY homolog response regulators. Ganusova et al. show that, in addition to fine-tuning the rotational bias of the polar flagellum, each of the four CheY homologs plays a distinct role in swimming (planktonic movement), swarming (collective movement across a surface), attachment to abiotic and biotic surfaces, and biofilm formation.

Finally, the emergence of the stator unit as a crucial component in sensing and motor modulation is underlined by three articles here. Islam et al. turn to the longstanding open question of the mechanism of stator ion selectivity and which ion holds energetic primacy for the BFM. They reconstructed and engineered ancestral sequences of the MotB stator sub-unit, and by assessing motor functionality as a function of the ionic power source, they add to our understanding of the contributions of specific residues within the ion channel. Biquet-Bisquert et al. sprovide a comprehensive review of the complex relationship between the ion motive force across the cell membrane, motor activity, and the dynamic assembly of stator units. Lin et al. use a fast perfusion microfluidic system to reveal that stator assembly dynamics in sodium-driven chimeric BFMs show a complex dependence upon the extracellular sodium concentration. Their results call into question current models of stator unit assembly and underline the extent to which the mechanisms of stator unit exchange remain unclear. These works reinforce the versatility of the BFM, as both a functional motor and as a bellwether of the bacterial environment, coordinating signals and response, to drive survival and spread.

## Author Contributions

All authors listed have made a substantial, direct, and intellectual contribution to the work and approved it for publication.

## Conflict of Interest

The authors declare that the research was conducted in the absence of any commercial or financial relationships that could be construed as a potential conflict of interest.

## Publisher's Note

All claims expressed in this article are solely those of the authors and do not necessarily represent those of their affiliated organizations, or those of the publisher, the editors and the reviewers. Any product that may be evaluated in this article, or claim that may be made by its manufacturer, is not guaranteed or endorsed by the publisher.

